# Predicting bird phenology from space: satellite‐derived vegetation green‐up signal uncovers spatial variation in phenological synchrony between birds and their environment

**DOI:** 10.1002/ece3.1745

**Published:** 2015-10-19

**Authors:** Ella F. Cole, Peter R. Long, Przemyslaw Zelazowski, Marta Szulkin, Ben C. Sheldon

**Affiliations:** ^1^ Department of ZoologyEdward Grey InstituteUniversity of OxfordOxfordUK; ^2^ Department of ZoologyBiodiversity InstituteUniversity of OxfordOxfordUK; ^3^ Environmental Change InstituteUniversity of OxfordOxfordUK; ^4^ Centre of New TechnologiesUniversity of WarsawWarsawPoland; ^5^ Centre d'Ecologie Fonctionnelle et EvolutiveUMR 5175 Campus CNRSMontpellierFrance

**Keywords:** Blue tit, environmental heterogeneity, EVI, great tit, mismatch, MODIS, reproductive phenology, satellite derived, spatial scale, wild population

## Abstract

Population‐level studies of how tit species (*Parus* spp.) track the changing phenology of their caterpillar food source have provided a model system allowing inference into how populations can adjust to changing climates, but are often limited because they implicitly assume all individuals experience similar environments. Ecologists are increasingly using satellite‐derived data to quantify aspects of animals' environments, but so far studies examining phenology have generally done so at large spatial scales. Considering the scale at which individuals experience their environment is likely to be key if we are to understand the ecological and evolutionary processes acting on reproductive phenology within populations. Here, we use time series of satellite images, with a resolution of 240 m, to quantify spatial variation in vegetation green‐up for a 385‐ha mixed‐deciduous woodland. Using data spanning 13 years, we demonstrate that annual population‐level measures of the timing of peak abundance of winter moth larvae (*Operophtera brumata*) and the timing of egg laying in great tits (*Parus major*) and blue tits (*Cyanistes caeruleus*) is related to satellite‐derived spring vegetation phenology. We go on to show that timing of local vegetation green‐up significantly explained individual differences in tit reproductive phenology within the population, and that the degree of synchrony between bird and vegetation phenology showed marked spatial variation across the woodland. Areas of high oak tree (*Quercus robur)* and hazel (*Corylus avellana*) density showed the strongest match between remote‐sensed vegetation phenology and reproductive phenology in both species. Marked within‐population variation in the extent to which phenology of different trophic levels match suggests that more attention should be given to small‐scale processes when exploring the causes and consequences of phenological matching. We discuss how use of remotely sensed data to study within‐population variation could broaden the scale and scope of studies exploring phenological synchrony between organisms and their environment.

## Introduction

Over recent decades there has been an increasing interest in plant and animal phenology as global warming has caused many life‐history events to shift earlier in time (Parmesan and Yohe 2003; Root et al. 2003; Durant et al. 2007). In temperate environments, characterized by transient peaks in resource availability, timing of reproductive events is a key determinant of reproductive success (Verhulst and Tinbergen 1991) and may be under strong selection (van Noordwijk et al. 1995). Research that has explored the mechanisms underlying the advancement of reproductive phenology over time often finds that phenotypic shifts can be explained by individual plasticity alone (Réale et al. 2003; Charmantier et al. 2008; Gienapp et al. 2008). Indeed, there is little evidence that current changes in climate have caused any evolutionary response within natural populations (reviewed in Merilä and Hendry 2014). While some species and populations are successfully tracking the shifting peak in their food source, others are not, leading to disruption of the synchrony between different trophic levels (Visser et al. 1998; Thackeray et al. 2010). One potential cause of this is that climate change can be temporally heterogeneous; for example, temperatures early in the spring when animals make decisions about timing of breeding may advance at a different rate to temperatures later in the season influencing the timing of food abundance (i.e., the selective environment) (Durant et al. 2007). To better understand these patterns of synchrony and asynchrony between trophic levels, we require a better knowledge of the factors influencing reproductive phenology.

One issue that hampers progress in this field is that insufficient attention has been paid to considering environmental phenology at a scale that is relevant to individual animals. Most studies exploring reproductive phenology to date have treated the environment as equivalent for all individuals within a population (e.g., Réale et al. 2003; Charmantier et al. 2008; Moyes et al. 2011). For example, a single site may be used to derive measures of temperature, or vegetation phenology, which is then related to measures of phenotype across a broader scale (e.g., an entire population). This approach implicitly assumes that environmental cues, to which the organisms respond, are invariant over quite large spatial scales. In reality, animals generally use a restricted amount of space within the range of a population, and significant environmental heterogeneity can mean that individuals with neighboring ranges experience quite different environmental conditions to one another (Hinks et al. 2015). However, collecting environmental data at a scale that is relevant to the individual animal for entire populations is likely to be highly labor‐intensive, is difficult to scale up, and usually cannot be carried out retrospectively.

One potential solution to this problem is to use remotely sensed satellite data to characterize biophysical properties of environments. Over recent decades, there has been a burgeoning interest in the ecological applications of remote‐sensing data (Roughgarden et al. 1991; Kerr and Ostrovsky 2003; Pettorelli et al. 2005, 2011; Cleland et al. 2007; Smith et al. 2013). Multispectral data, collected by optical sensors on‐board satellites orbiting the earth, can be used to calculate indices of vegetation primary productivity (Reed et al. 1994). The normalized difference vegetation index (NDVI) and the enhanced vegetation index (EVI) are two such measures, which take advantage of the fact that the relative amount of red and near infrared light reflected back to the satellite depends on vegetation cover. Some satellite remotely sensed datasets are available on a global scale, are often free of charge, and are increasingly provided preprocessed and having undergone some degree of quality control. This wealth of data is increasingly being used to gain insight into vegetation dynamics, biodiversity, animal movements, and population dynamics (reviewed in Pettorelli et al. 2005, 2011; Hurley et al. 2014).

The different satellite datasets vary in both their spatial and temporal resolution. When images of the same site are collected frequently enough, they can be used to quantify vegetation phenology measures such as the timing of spring green‐up (e.g., Soudani et al. 2008; Busetto et al. 2010; Hufkens et al. 2012). Such data have been used on a continental scale to show that large‐scale spatial patterns of the onset of plant growth correlate with spatial variation in spring warmth (Chen et al. 2005). They have also been used on a smaller scale to explore trophic interactions between vegetation and consumers. For example, one study on reindeer (*Rangifer tarandus*) reported negative correlations between vegetation green‐up and both calf mass and female reproductive success (Tveraa et al. 2013). To date, most studies that have explored how satellite‐derived vegetation measures relate to life‐history traits of higher trophic levels do so at the population level, either comparing populations to one another or the same population across years (but see Szulkin et al. 2015). However, the increasing availability of fine spatial (e.g., SPOT, Landsat, or the recently launched Sentinel‐2a) and temporal (e.g., MODIS, future Sentinel 2 constellation) resolution of satellite sensors offers considerable potential for individual‐based studies, which is yet to be fully explored.

In this study, we focus on the tri‐trophic system of passerine songbirds feeding on caterpillar prey that in turn feed on the emergent leaves of deciduous tree (see Visser et al. 1998; Charmantier et al. 2008), to explore whether satellite data can be used as an appropriate proxy for spatiotemporal variation in resource abundance. Great tits (*Parus major*) and blue tits (*Cyanistes caeruleus*) primarily rear their offspring on lepidopteran larvae, particularly of the winter moth (*Operophtera brumata*). These caterpillars are only available during a brief period in spring as they time their hatching to exploit newly emerged leaves of their host trees (particularly oaks *Quercus* spp.), and then pupate in the soil. In order to maximize their reproductive success, tits must time their breeding so that their peak in food demand, which comes when nestlings are approximately one week old, coincides with the transient peak in caterpillar abundance (Verhulst and Tinbergen 1991). Onset of laying is considerably plastic, as individual great tits have been shown to track the phenology of their food source over their lifetime (Charmantier et al. 2008). In temperate deciduous woodlands throughout the Northern Hemisphere, tree phenology is largely determined by winter chilling followed by warming in spring (Hunter and Lechowicz 1992), but there is also marked within‐population variation at a scale that is relevant to individual birds (Hinks et al. 2015). To date, this fine‐scale spatial component has largely been ignored in studies of tit phenology (but see Nager and van Noordwijk 1995).

Here, we used great tit and blue tit breeding data and satellite imagery over a 13‐year period to determine whether between and within‐year variation in reproductive phenology for a single population can be predicted by satellite‐derived vegetation phenology. We judged vegetation phenology to be a suitable proxy for caterpillar phenology as previous work has shown that green‐up measures derived from MODIS data can be a good indicator of the onset of oak bud‐burst in deciduous woodlands (Soudani et al. 2008), and that the timing of oak budburst at the level of individual trees is a strong predictor of peak in caterpillar availability (Hinks et al. 2015). We used a Moderate Resolution Imaging Spectroradiometer (MODIS) satellite data product, which has a spatial resolution of 240 m and a temporal resolution of 8 days, to derive nestbox‐specific vegetation “green‐up” dates for 1207 nestboxes in a 385‐ha mixed‐deciduous woodland. Our aims in this study were threefold. First, we tested whether annual measures of vegetation green‐up for our study site can be used to predict between‐year variation in the timing of the annual peak in caterpillar abundance and the timing of great tit and blue tit breeding. Second, we quantified the relationship between local vegetation green‐up and tit phenology at the level of individual breeding attempts within years. Finally, we explored whether the strength of this synchrony between tits and their environment varies across the study site, and what may explain this variation, and test how these patterns differed between the two tit species. In addition, we investigated whether the amount of cloud cover over the study site influenced the predictive power of the MODIS satellite data to explain tit reproductive timing.

## Materials and Methods

### Study site and population

This study was carried out using a nestbox population of great tits and blue tits at Wytham Woods (51°46′N, 1°20′W), a mixed‐deciduous 385‐ha woodland in Oxfordshire. The canopy composition is primarily oak (*Quercus robur*), ash (*Fraxinus excelsior*), sycamore (*Acer pseudoplatanus*), and beech (*Fagus sylvatica*), with an understory of hawthorn (*Crataegus momgyna*), hazel (*Corylus avellana*), elder (*Sambucus niger*), and field maple (Acer *campestre*) (Perrins 1965). All live oak trees in Wytham with a diameter at breast height of 30 cm of greater (*N* = 5425) were mapped using handheld GPS during February 2011. Wytham is divided into 121 discrete compartment bounded by paths and linear features (mean area = 3.15 ha, standard deviation = 2.90, see Figure S1). At the same time that the oaks were mapped, habitat data were collected for each of the 121 compartments. For each compartment, the proportion of canopy that was composed of each tree species (oak, ash, beech, birch, sycamore, cedar, field maple, fir, hemlock, larch, poplar, pine, spruce, sweet chestnut, willow, other) was estimated by eye, as well as the percentage of overall canopy cover. Similar data were collected for the understory layer (species: hazel, hawthorn, blackthorn, birch, elder, field maple, holly and willow, other). These measures were estimated by experienced forestry ecologists.

Great tit and blue tit breeding data spanning 13 years (2001–2013) were collected as part of the long‐term study of tits in Wytham (see McCleery et al. 2004; Wood et al. 2007). We used this timespan because full monitoring of blue tit nests did not begin until 2001. In order to compare effects in the two species, we therefore restricted the great tit data to 2001–2013. There are 1207 nestboxes in Wytham, the locations of which were digitally mapped using a differential GPS system (Wilkin et al. 2007); 1020 of these are standard boxes used by both tit species (entrance hole diameter: 32 mm), and the remaining 187 have smaller entrance holes (diameter: 26 mm), which prevent great tits from entering. During April and May, all nestboxes are visited weekly to monitor breeding attempts. Tits generally lay one egg a day for approximately 8 days (Perrins 1965), and therefore, the date when the first egg is laid in a nest (hereafter laying date) can be inferred by counting backwards from the date that the clutch was observed (e.g., a nest with 3 eggs on 3rd April would have a laying date of 1st April). Hatch date is ascertained by visiting all nests on alternate days from the eleventh day after clutch completion until hatching. Parents were captured at the nest while feeding 8‐ to 12‐day‐old nestlings and ringed with a unique metal leg ring or identified if already ringed. Age and sex of parents were established on the basis of plumage characteristics (Svensson 1994). Only clutches laid within 30 days of the first clutch of the year were used in this study to exclude second breeding attempts by individuals (van Noordwijk et al. 1995).

### Caterpillar data

Timing of the population peak in winter moth larvae abundance is measured every year as part of a long‐term study (see Charmantier et al. 2008). The half‐fall date (the date by which 50% of the seasonal total of larvae has descended to the ground to pupate) was determined for the same four oak trees at a single site in Wytham. This was done by placing two water traps, 100 × 50 cm in size, under each tree. Traps were visited regularly throughout the spring to count and remove fallen winter moth larvae. These data give an indication of the relative timing of the population peak of caterpillar biomass and are strongly correlated with annual variation in great tit timing of breeding (Charmantier et al. 2008).

### Satellite data

We used freely available data from the MODIS sensors (NASA 2008) to determine vegetation spring green‐up dates for Wytham. These sensors are on‐board Terra and Aqua satellites, which were launched in 1999 and 2002, respectively, and acquire multispectral images over the whole Earth surface every 1 to 2 days. All pixels covering Wytham (*n* = 117, see Supplementary Methods and Figure S2) were projected to 240 m resolution on WGS 1984 UTM 30N coordinate system. The enhanced vegetation index 2 (EVI2) (Jiang et al. 2008) was then calculated for each pixel in each 8‐day period. For each 240 m pixel and 8‐day period, there were potentially 16 (8 am and 8 pm) available clear cloud‐free observations of the land surface. However, in practice, because of cloud, typically only approximately 4 clear observations of the land surface were available for each 240 m pixel in each 8‐day period. Observations are flagged as “cloudy” or “cloud‐free” by the MOD03 cloud‐detection algorithm. EVI2 on the middle day of each 8‐day period was then estimated as the mean of all available clear observations in the 8‐day period. Very rarely (<0.5%), no clear observations were available for a particular 240 m pixel in an 8‐day period and it was not possible to estimate EVI2 for that time period.

**Figure 1:**
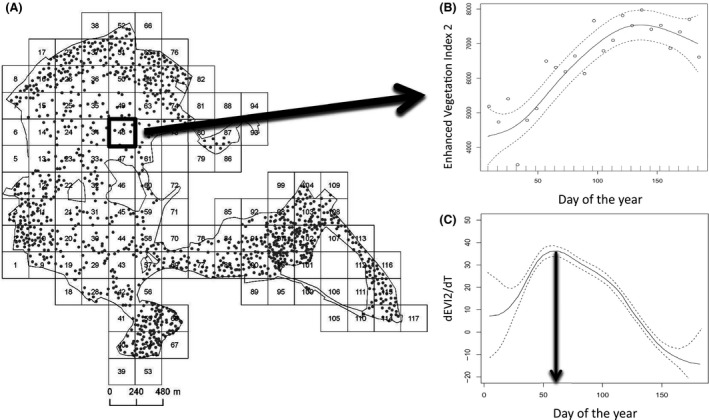
Method for calculating pixel‐specific vegetation green‐up dates: (A) 117 MODIS pixels (240 m × 240 m) which intersect with Wytham (gray dots show nestboxes); enhanced vegetation index was calculated for each pixel for every 8‐day period, (B) an example generalized additive model (GAM) curve fitted to the EVI2 data for a single pixel, and (C) plot showing the change in EVI2 over time for the same data, the arrow indicates maximum rate of change, and the vegetation green‐up date. Dashed lines in (B) and (C) show 95% confidence intervals.

There are many methods of calculating spring phenological transition dates from time series of multispectral images for deciduous woodlands (Jönsson and Eklundh 2004), mostly corresponding to the date at which the rate of change in curvature in a vegetation index (VI) exhibits local minima or maxima (Zhang et al. 2003). The inflexion point of the piece‐wise logistic curve fitted to VI data (i.e., the point of maximum rate of change) has been shown to be a good indicator of the onset of oak bud‐burst in deciduous woodlands (Soudani et al. 2008). In this study, we used generalized additive models (GAMs; Hastie and Tibshirani 1990) to calculate a very similar metric, which were selected because we did not want to impose any prior assumption on the shape of the green‐up curve. A GAM was fitted to the EVI2 data for each pixel individually (EVI2 vs. day of year). The day of vegetation green‐up for each pixel was calculated as the day on which the derivative of this function was maximized (i.e., the day of the greatest rate of change in green‐up per year, see Figure 1). GAMs were fitted using the R package “mgcv” version 1.8‐4 and differentiated using the function predict.gam of mgcv (Wood 2011).

Nestbox‐specific green‐up dates were calculated in ArcGIS 10.2 using a weighting procedure to account for where in a pixel a nestbox was located, as well as what proportion of a pixel was made up of nonwoodland habitat (see Supplementary Methods and Figure S2 for further details).

### Cloud cover data

We analyzed information on temporal patterns of cloudiness based on the same MODIS sensors as in the case of EVI data, this time using the MOD08 product (collection 5.1, accessed through Level 1 and Atmosphere Archive and Distribution System – LAADS, ladsweb.nascom.nasa.gov), which aggregates data into 8‐day statistics, and a 1° grid. This was done to explore whether cloud cover influenced the predictive power of the MODIS satellite data to explain tit reproductive timing. This could conceivably be the case given that cloud will obscure the ground, reducing the number of valid measurements forming the time series used to describe each pixel's green‐up trajectory. This in turn will reduce the confidence in the green‐up date estimates. In order to determine the key time period of cloudiness that may influence the relationship between MODIS vegetation green‐up and tit reproductive phenology, we ran a series of linear regressions testing the relationship between how well tits matched nestbox‐specific green‐up each year and early spring cloudiness (cloud fraction (%)) averaged over different time windows. For each year, the correlation coefficient for the relationship between individual laying date and nestbox‐specific green‐up was calculated, providing an estimate of annual synchrony between tit and vegetation phenology. The cloudiness data had a temporal resolution of 8 days. Average cloudiness was calculated for the 91 different time windows, which ranged between 24 days (3 8‐day periods) and 120 days (15 8‐day periods) in length, within the first 120 days of the year. Linear regressions were used to test the relationship between the annual tit vs. green‐up correlation coefficients and each of the 91 sets of cloudiness measures (*N* = 13 years for each regression). These models were then ordered by *r*‐value. The top models for both great tits and blue tits were highly significant (see Table 4 and Fig. 7). In addition, we analyzed data for the whole of Europe in order to quantify cloudiness in areas and seasons relevant to the type of research considered in this study.

### Statistical methods

All analyses were run on the great tit dataset and the blue tit dataset independently. To address our first objective, we used linear models to test the relationship between annual green‐up dates (average green‐up date for all pixels containing more than 50% woodland) and (1) annual caterpillar half‐fall date; (2) annual mean laying date and (3) annual mean hatching date (*n* = 13 years). Linear mixed models (LMMs) were used to address our second objective, exploration of the relationship between within‐year individual variation timing of breeding (laying dates and hatch dates) and nestbox‐specific green‐up dates. Green‐up dates were partitioned into between‐year variation (annual mean green‐up date) and within‐year variation (deviation from annual mean green‐up date), following methods from van de Pol and Wright (2009), to control for between‐year effects. We first ran minimal models containing no additional fixed effects to determine the strength of the relationship between vegetation green‐up and timing of breeding. We then ran models that controlled for three known environmental correlates of laying date in our population: altitude (in meters), distance to the edge of the woodland (in meters), and local oak density (number of oak trees within 75 m of a nestbox: see Wilkin et al. 2007). These environmental factors may themselves explain a portion of the variation in green‐up at any given location – for example, vegetation green‐up is likely to be later at higher altitudes. We therefore wanted to test whether vegetation green‐up explained variation in timing of breeding above and beyond that explained by these known environmental factors. All models also included the random effects of nestbox ID and year. Ideally, female identity should be controlled for in these analyses to account for individuals that breed in successive years being represented in the dataset more than once. However, we did not included this effect in our main models because a significant proportion of breeding attempts failed before the associated female could be identified or the female was not successfully caught. It was inappropriate to exclude these breeding attempts from the analysis because they represented a biased sample of laying dates. Identified females began laying significantly earlier than unidentified females (mean laying date ± SE: great tits: female ID unknown: 21.66 ± 0.27, female ID known: 21.06 ± 0.12, LM: *t* = −2.06, *P* = 0.039, *n* = 4597; blue tits: female ID unknown: 20.68 ± 0.21, female ID known: 19.21 ± 0.12, LM: *t* = −5.89, *P* < 0.001, *n* = 5221). However, we reran all analyses including female ID as a random factor and present results in the supplementary material (see Table S1). Local oak density was calculated using GIS software (ArcGIS 10.2), by mapping the locations of all oak trees and nestboxes and then calculating the number of oak trees within a 75 m buffer around each nestbox (see Wilkin et al. (2007) for details of methodology). A radius of 75 m was used because a previous study on the Wytham great tit population compared the predictive power of oak density within different sized buffers around each box to explain variation in laying date and found that 75 m explained the most variation (Wilkin et al. 2007).

To address our final objective – establishing whether the relationship between satellite‐derived green‐up and tit phenology varies across the woodland, for each pixel, we calculated the correlation coefficient quantifying the relationship between annual average laying date for that pixel and annual pixel green‐up date. This pixel‐level analysis was restricted to the 67 pixels that were comprised of 50% woodland or more (see Figure S2) in order to reduce the signal interference caused by farmland that borders the woodland. For the great tit analysis, three further pixels were excluded, one because it did not include any great tit breeding attempts, and two, because the correlation coefficients for these pixels were based on only three data points each (i.e., birds only bred in these pixels 3 of 13 years). The remaining 64 pixels each had between 9 and 13 data points (i.e., 9–13 years where great tits bred in a given pixel). Similarly for the blue tit analysis, two pixels were excluded because they were based on three and five data points. The remaining 65 pixels each had between 8 and 13 data points.

To test whether habitat type related to the degree of phenological synchrony between tit and vegetation phenology at the pixel level, we ran linear regressions with the pixel correlation coefficient (laying date vs. green‐up) as the response variable and fixed effects: proportion of pixel canopy comprised of ash, oak, beech, sycamore, and proportion of the understory comprised of hazel, hawthorn, elder, and field maple. These eight species are the dominant vegetation types in Wytham, accounting for 31%, 27%, 14%, and 11% of the canopy vegetation, respectively, and 44%, 33%, 7%, and 6% of the understory vegetation, respectively. Relationships between pixel‐level synchrony and abundance of each of the eight tree and shrub species were tested separately as their relative abundance was not independent of one another. Pixel‐level canopy and understory data were then calculated by overlaying the 240 m MODIS pixels and the habitat compartments (see study site section) using ArcGIS 10.2 software, and weighting compartment habitat values based on the proportion they contributed to the area of a given pixel. These models included all pixels composed of greater than 50% woodland and were weighted based on the total number of breeding attempts in each pixel to account for the variation in confidence in the pixel correlation coefficients. We examined the spatial structure in laying date and phenological synchrony using Mantel correlograms. All analyses were carried out using R version R 3.0.1 (http://www.R-project.org).

## Results

### Relating annual measures of vegetation, caterpillar, and tit phenology

**Figure 2:**
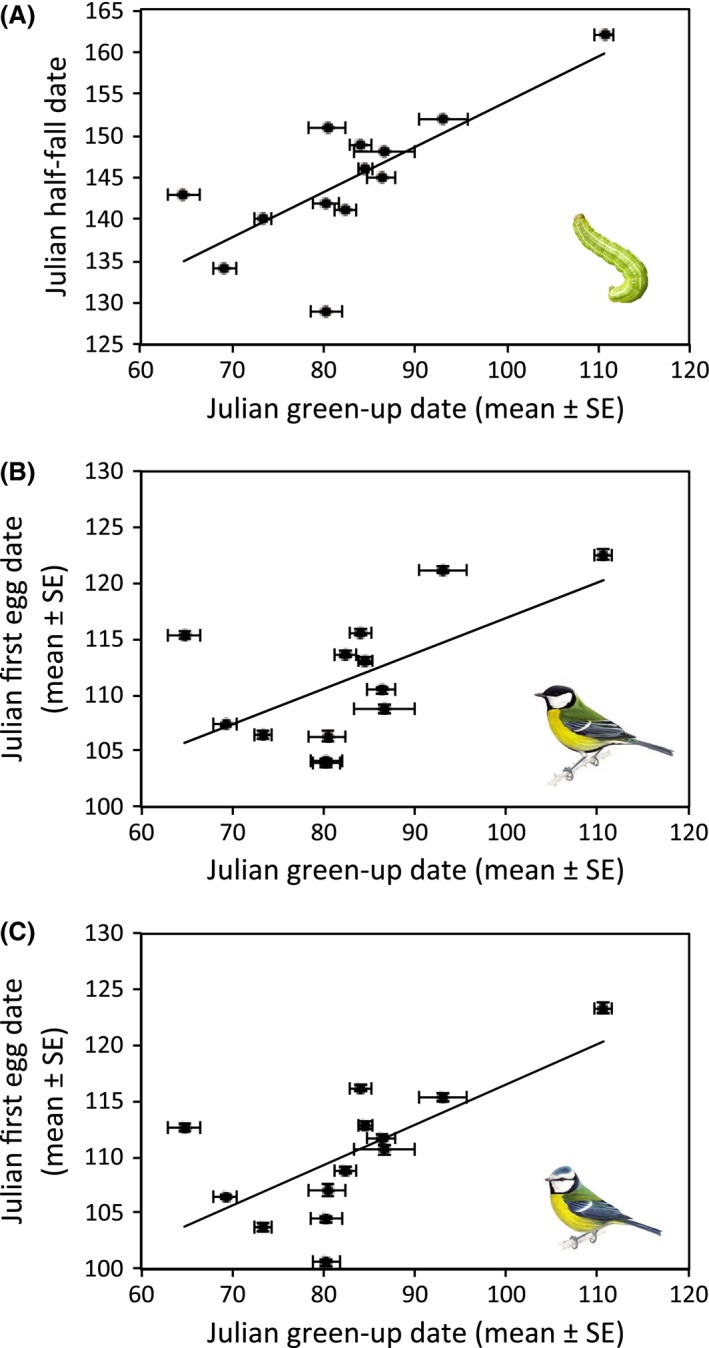
Correlations demonstrating the relationships between annual green‐up date (for the entire woodland) and (A) annual caterpillar half‐fall date (peak caterpillar abundance), (B) annual mean great tit laying date, and (C) annual mean blue tit laying date (error bars represent standard error of the mean).

Taking study site wide averages for the 13 years, vegetation green‐up date was positively correlated with caterpillar half‐fall date (*r* = 0.74, *P* = 0.004, *n* = 13, Fig. 2A) and with the population mean laying date and hatching date for great tits (*r* = 0.60, *P* = 0.032, *n* = 13; *r* = 0.61, *P* = 0.027, *n* = 13, respectively, Fig. 2B) and blue tits (*r* = 0.68, *P* = 0.010, *n* = 13; *r* = 0.67, *P* = 0.012, *n* = 13, respectively, Fig. 2C). Hence, at the scale of the entire woodland, this measure detects annual variation linked to the phenology of other trophic levels.

### Relating satellite data to tit phenology at the individual level

**Figure 3:**
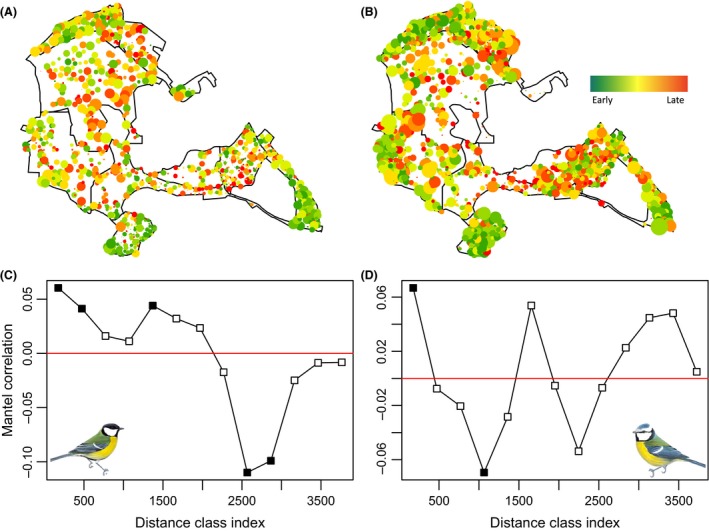
Spatial variation in mean laying date for all nestboxes 2001–2013 for (A) great tits and (B) blue tits. Dot size represents the number of years each box was occupied. As the blue tit breeding attempts were spread over a greater number of nestboxes (see methods section), dot size for blue tits is enlarged (×1.5) for illustrative purposes. Correlogram demonstrating positive and negative spatial autocorrelation at multiple spatial scales for (C) great tits and (D) blue tits (run on nestboxes occupied 7 or more times). Closed squares indicate significant autocorrelation (*P* < 0.05) and open squares nonsignificant values. Data corrected for annual and female‐specific effects.

**Figure 4:**
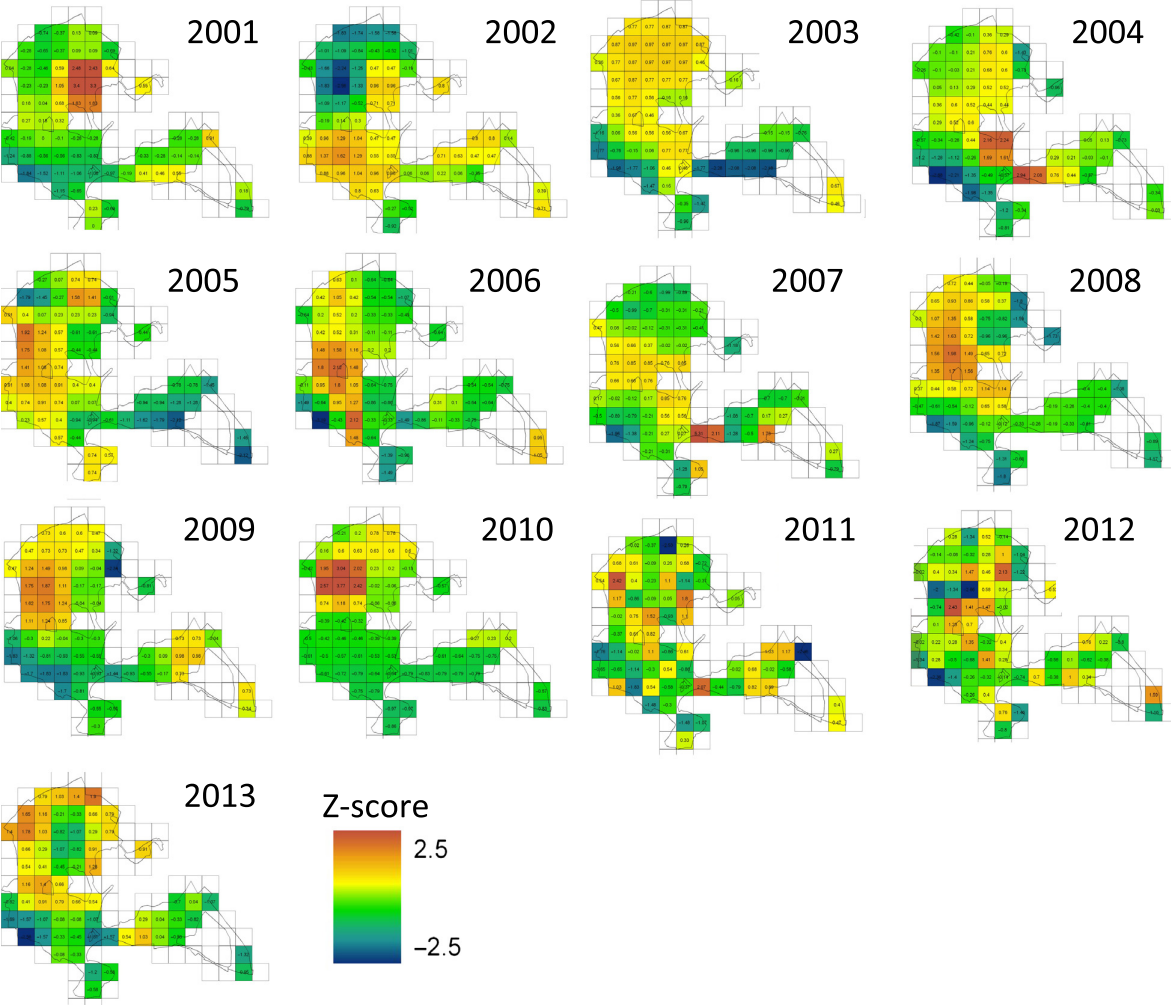
Spatial variation in spring vegetation green‐up date (2001–2013), where green‐up for each pixel was calculated as the day of the greatest rate of change in green‐up, derived from a time series of MODIS satellite images for each pixel with >50% woodland (*n* = 67). Green‐up date is *z*‐transformed within year (centered using annual mean and divided by annual standard deviation, range ‐2.5–2.5).

The timing of onset of laying in great tits and blue tits varied across the study site (see Fig. 3A and B) analysis of spatial autocorrelation in these data (Mantel r) reveals both positive and negative autocorrelation depending on the scale, characteristic of data aggregated into patches of similar observations (Fig. 3C and D). There was also substantial variation in vegetation green‐up data both within and between years (Fig. 4 and Figure S3). At the level of individual breeding attempts, nestbox‐specific green‐up date was related to both onset of laying and hatching date in both great tits (laying date: *t* = 3.975, pMCMC<0.001, B ± SE = 0.027 ± 0.007, *n* = 4597; hatching date: *t* = 4.845, pMCMC < 0.001, B ± SE = 0.032 ± 0.007, *n* = 4065) and blue tits (laying date: *t* = 2.537, pMCMC = 0.012, B ± SE = 0.018 ± 0.007, *N* = 5003; hatching date: *t* = 3.057, pMCMC < 0.001, B ± SE = 0.020 ± 0.007, *n* = 4405). In great tits, these relationships remained significant, albeit weaker, when controlling for altitude, distance from the edge of the woodland, and local oak density, which were all significantly related to both laying date and hatch date (see Table 1a). Hence, satellite‐derived vegetation phenology is informative, even when likely environmental effects such as those due to altitude are controlled for. In blue tits, however, once these other environmental factors were included, green‐up date became nonsignificant at the *P* = 0.05 level (*P* = 0.124 and *P* = 0.066 for laying date and hatching date, respectively, see Table 1b). Given the large sample size, such residual effects are weak. Including female ID as a random factor, which excludes nests that failed before the female could be identified and those where the female could not be caught, yielded very similar results (see Table S1).

**Table 1:** Model outputs from linear mixed models testing the relationship between individual laying date and hatch date and nestbox‐specific green‐up date for (a) great tits and (b) blue tits

(a) Great tit	Laying date (*n* = 4597)				Hatch date (*n* = 4065)			
	Coefficient	SE	*t* value	pMCMC	Coefficient	SE	*t* value	pMCMC
Intercept	−7.384	10.390	−0.711		12.826	9.731	1.318	
Green‐up (within‐year)	0.019	0.007	2.887	<0.001	0.024	0.007	3.612	0.002
Green‐up (between‐year)	0.315	0.124	2.542	0.03	0.328	0.116	2.823	0.014
Altitude	0.028	0.003	8.357	<0.001	0.028	0.003	8.379	<0.001
Distance from woodland edge	0.003	0.001	3.107	0.002	0.005	0.001	5.543	<0.001
Local oak density	−0.043	0.006	−7.335	<0.001	−0.034	0.006	−5.783	<0.001

(b) Blue tit	Laying date (*n* = 5003)				Hatch date (*n* = 4405)			
	Coefficient	SE	*t* value	pMCMC	Coefficient	SE	*t* value	pMCMC
Intercept	−9.963	9.394	−1.061		11.802	9.537	1.237	
Green‐up (within‐year)	0.011	0.007	1.579	0.124	0.012	0.007	1.785	0.066
Green‐up (between‐year)	0.334	0.112	2.975	0.010	0.341	0.114	2.986	0.014
Altitude	0.035	0.003	10.009	<0.001	0.037	0.003	11.636	<0.001
Distance from woodland edge	0.000	0.001	0.406	0.636	0.004	0.001	4.815	<0.001
Local oak density	−0.063	0.005	−11.959	<0.001	−0.042	0.005	−8.598	<0.001

Models control for the fixed effects: altitude, distance from the edge of the woodland and local oak density, and random effects: year and nestbox. Variation in green‐up date was partitioned into within‐ and between‐year variation (following van de Pol and Wright 2009).

### Spatial variation in phenological synchrony

**Figure 5:**
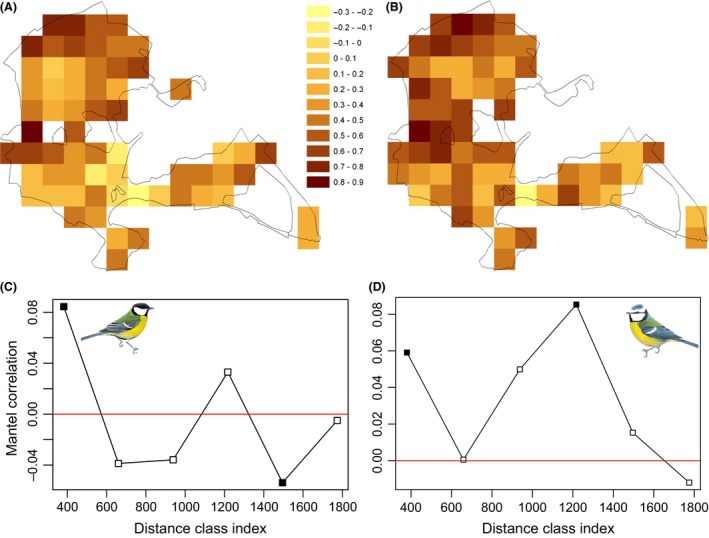
Spatial variation in phenological matching 2001–2013 for (A) great tits (*n* = 64 pixels) and (B) blue tits (*n* = 65 pixels). Pixel color represents the strength of matching (yellow‐red denotes low to high correlation between vegetation green‐up and tit laying date). The correlation coefficient for each pixel was calculated by plotting the mean annual laying dates against annual pixel green‐up scores. Correlogram demonstrating positive and negative spatial autocorrelation in phenological matching in (C) great tits and (D) blue tits. Closed squares indicate significant autocorrelation (*P* < 0.05) and open squares nonsignificant values.

The extent to which satellite‐derived green‐up date predicted timing of breeding across years varied markedly across the woodland for both great tits and blue tits (Fig. 5A and B), ranging from *r* = −0.31 to *r* = 0.82 for great tits and *r* = −0.29 to *r* = 0.87 for blue tits. At the pixel level, there was a strong positive correlation between great tit and blue tit synchrony with green‐up (*r* = 0.64, *P* < 0.001, *n* = 61), and overall, blue tits were significantly more synchronized with green‐up than great tits (*t* test: *t* = 2.245, *P* = 0.027, blue tit mean pixel correlation coefficient ± SE: 0.457 ± 0.026, *n* = 65; great tit mean pixel correlation coefficient ± SE: 0.367 ± 0.030, *n* = 64). There was also significant spatial autocorrelation in these data, with neighboring pixels being more similar to each other (in terms of trophic synchrony across years) than those that were further apart (Fig. 5C and D).

Forest composition significantly explained variation in phenological synchrony in both great tits and blue tits. In both species, pixels where tit and vegetation phenology were strongly correlated were significantly more oak‐rich compared to poorly synchronized pixels (see Table 2a and b). In blue tits, the proportion of the canopy comprised of ash was also significantly related to synchrony, with ash‐rich pixels being less synchronized than ash‐poor pixels (see Table 2b). This relationship was not present in the great tit data, and neither of the other two dominant canopy species (beech and sycamore) significantly predicted phenological synchrony at the pixel level in either tit species (see Table 2).

**Table 2:** Model outputs from linear regressions testing the relationship between how well tits matched pixel‐level green‐up and canopy composition for (a) great tits (*n* = 64) and (b) blue tits (*n* = 65)

	Coefficient	SE	*t* value	*P*
(a) Great tits				
Oak	0.394	0.121	3.253	0.002
Ash	0.073	0.145	0.499	0.620
Beech	−0.319	0.182	−1.751	0.085
Sycamore	−0.374	0.212	−1.764	0.083
(b) Blue tits				
Oak	0.395	0.112	3.521	0.001
Ash	−0.267	0.117	−2.291	0.025
Beech	0.015	0.197	0.074	0.941
Sycamore	−0.372	0.214	−1.740	0.087

Habitat measures (proportion of pixel canopy comprised of oak, ash, beech, and sycamore) were each tested independently.

Of the four understory species, only hazel and elder significantly related to synchrony. In both species, synchrony with green‐up was higher in hazel‐rich pixels, but this relationship was considerably weaker in blue tits (see Table 3). Additionally, in both species, the abundance of elder was negatively correlated to phenological synchrony (see Table 3). Neither of the other two dominant understory species (hawthorn and field maple) significantly predicted phenological synchrony at the pixel level in either tit species.

**Table 3:** Model outputs from a linear regressions testing the relationship between how well tits matched pixel level green‐up and understory composition for (a) great tits (*n* = 64) and (b) blue tits (*n* = 65)

	Coefficient	SE	*t* value	*P*
(a) Great tits				
Hazel	0.261	0.089	2.934	0.005
Hawthorn	−0.125	0.096	−1.306	0.196
Elder	−0.643	0.255	−2.520	0.014
Field maple	−0.429	0.472	−0.909	0.367
(b) Blue tits				
Hazel	0.163	0.081	2.016	0.048
Hawthorn	−0.088	0.090	−0.987	0.327
Elder	−0.536	0.237	−2.260	0.027
Field maple	−0.160	0.443	−0.361	0.719

Habitat measures (proportion of pixel understory comprised of hazel, hawthorn, elder, and field maple) were each tested independently.

### Cloud cover

**Figure 6:**
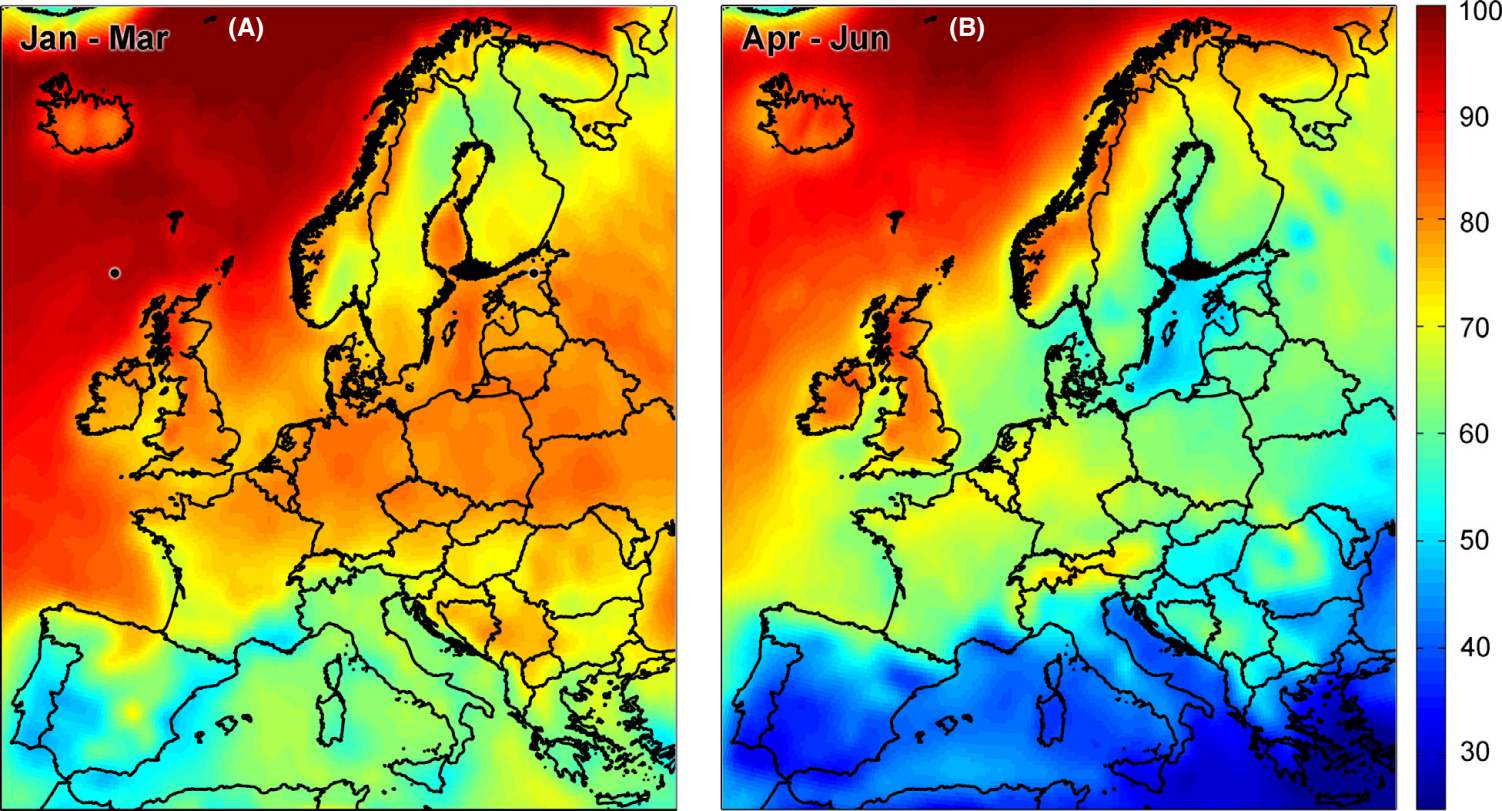
Average cloud fraction (%, MODIS sensors) across Europe from (A) January to March and from (B) April to June (2001–2013).

**Figure 7:**
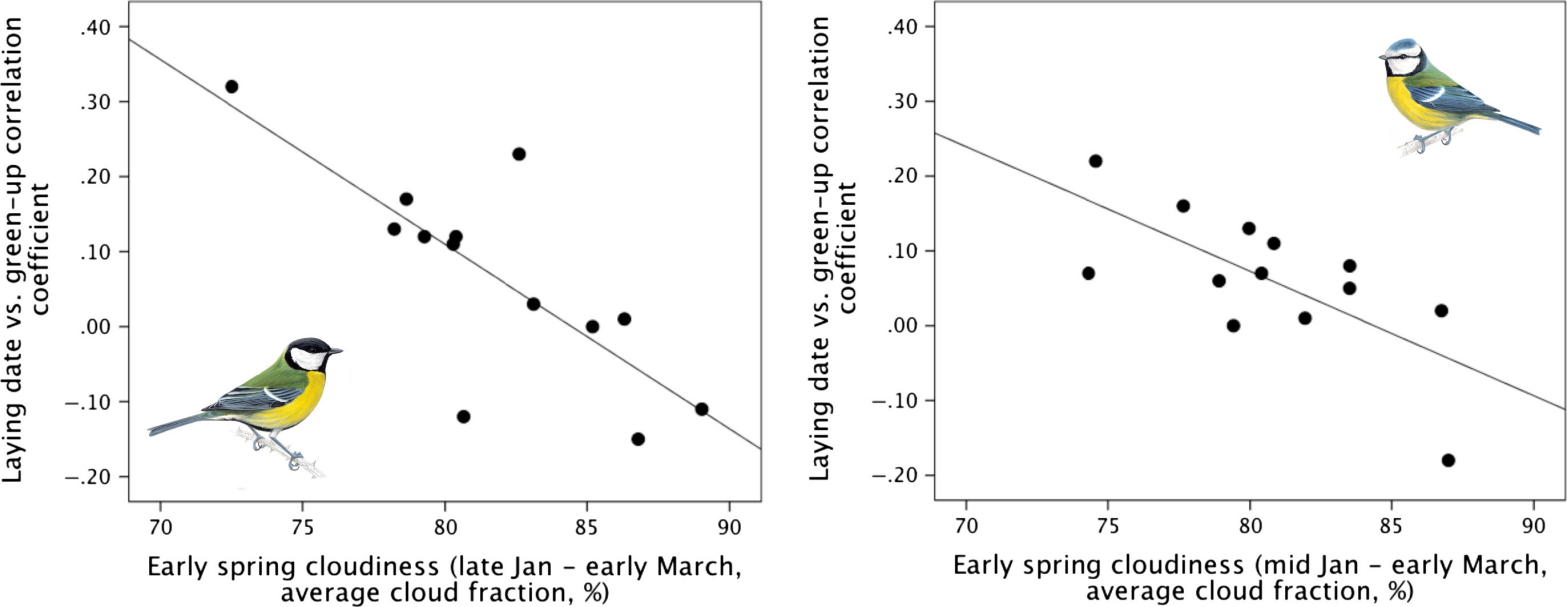
Correlations between how well tits matched nestbox‐specific green‐up each year and early spring cloudiness for (A) great tits (*r* = 0.77**) and (B) blue tits (*r* = 0.69**).

Cloudiness varied considerably across Europe, with the UK experiencing higher than average cloudiness relative to continental Europe (Fig. 6). For Wytham, cloudiness varied both across and between years (see Figures S4 and S5). At the level of years, cloudiness was strongly related to the strength of the correlation between vegetation green‐up and tit timing of breeding (Fig. 7). The period of timing for which this relationship was the strongest was late January – early March in great tits (*r* = 0.774) and mid‐January to early March in blue tits (*r* = 0.689, see Table 4).

**Table 4:** Model outputs from linear regressions testing the relationship between an annual measure of how well tits matched nestbox‐specific green‐up and spring cloudiness for (a) great tits and (b) blue tits

(a) Great tit						January				February				March				April		
	Coefficient	SE	*t*	*P*	*r*	01	02	03	04	05	06	07	08	09	10	11	12	13	14	15
Intercept	2.079	0.497	4.183	0.002																
Time block 4–9	−0.025	0.006	−4.055	0.002	0.774															
Intercept	2.233	0.545	4.102	0.002																
Time block 5–9	−0.026	0.007	−3.985	0.002	0.769															

(b) Blue tit						January				February				March				April		
	Coefficient	SE	*t*	*P*	*r*	01	02	03	04	05	06	07	08	09	10	11	12	13	14	15
Intercept	1.404	0.427	3.290	0.007																
Time block 2–9	−0.017	0.005	−3.149	0.009	0.689															
Intercept	1.350	0.430	3.142	0.009																
Time block 5–9	−0.016	0.005	−3.002	0.012	0.671															

The top two models with the highest r‐value are shown for each species. The cloudiness data (cloud fraction (%)) have a temporal resolution of 8 days. Average cloudiness was calculated for all 91 different time windows, which range between 24 days (3 8‐day periods) days to 120 days (15 8‐day periods) in length, within the first 120 days of the year. The right‐hand panel illustrates the time periods covered by the top models (shaded in grey).

## Discussion

Remotely sensed data are rapidly becoming a key resource in ecological research. Measures of vegetation productivity have proved especially useful, with new uses arising continuously (Kerr and Ostrovsky 2003; Pettorelli et al. 2011). These measures can be used to quantify the spatial and temporal aspects of vegetation development, which in turn can be used to predict the population dynamics and life‐history traits of both primary and secondary consumers. Here, we investigate whether satellite‐derived measures of vegetation phenology can be used to predict individual variation in the timing of laying in a blue tit and a great tit population occupying the same woodland. We show that there is significant spatial structure in reproductive phenology within these populations, and that this variation relates to satellite‐derived vegetation green‐up dates, calculated for each individual nest location. In great tits, this relationship remained significant after controlling for altitude, distance from the edge of the woodland and local oak density, which are environmental variables known to influence timing of breeding in tits (and which are likely to influence vegetation phenology). In contrast, timing of reproduction in blue tits was more strongly influenced by local habitat quality (oak density) and altitude, which is likely to be closely related to early spring temperature. Summarizing breeding attempts at the level of individual 240 m pixels, we demonstrate that the extent to which vegetation green‐up and timing of laying are synchronized varies spatially across the study site, and that this variation relates to both canopy and understory composition.

### Satellite‐derived vegetation green‐up: an appropriate proxy for tit food availability?

The use of remotely sensed vegetation data in ecology is often thought to mainly be applicable to herbivorous species (see Pettorelli et al. 2011), but there are in fact many studies that have successfully used these data to explore ecological questions in nonherbivorous birds and mammals (reviewed in Pettorelli et al. 2011; Hurley et al. 2014). This is possible in systems where the availability of prey for the species of interest is strongly influenced by vegetation characteristics. The tri‐trophic food chain of tits, caterpillars, and oak trees is an example of such a system. The peak in winter moth caterpillar biomass is highly dependent on the budburst date of oak trees, its favored host species (van Asch and Visser 2007). Indeed, a recent study in Wytham found a strong correlation (*r* = 0.67) between oak bud‐burst date and the date of the peak in caterpillar abundance at the level of individual trees (Hinks et al. 2015). Furthermore, the same study also found that individual differences in onset of laying by great tits could be predicted by the phenology of the nearest oaks to a nesting site (Hinks et al. 2015). These findings suggest that the timing of oak spring green‐up can be used as a reliable predictor for when nestling prey is most abundant in the environment.

In this study, we used vegetation green‐up dates derived from MODIS satellite data as a proxy for oak tree spring phenology, but acknowledge it is likely to capture green‐up of a range of understory and canopy species. A similar approach has been applied using MODIS's predecessor, Advanced Very High Resolution Radiometer (AVHRR), to relate advancing oak phenology to average timing of breeding in pied flycatchers (*Ficedula hypoleuca*) across years (Sanz et al. 2003). This study used data with a spatial resolution of 8 km and therefore could not look at within‐population variation in bird phenology. In addition to providing data with a higher spatial resolution, MODIS sensors collect data with a greater spectral resolution and have improved geo‐location accuracy and atmospheric corrections (Soudani et al. 2008). There are various different methods of calculating spring phenological transition dates for deciduous woodlands (see Jönsson and Eklundh 2004), mostly corresponding to the date at which the rate of change in curvature in a vegetation index (VI) exhibits local minima or maxima (Zhang et al. 2003). These include the date of onset of photosynthetic activity (Zhang et al. 2003), the date of maximum rate of change in VI (Soudani et al. 2008; Busetto et al. 2010), averaged VI in a fixed time window (Sanz et al. 2003), and the date corresponding to the half‐maxima of a sigmoid curve fitted to the vegetation data (Fisher et al. 2006). A recent study compared field measurements of tree bud‐burst to several different phenological green‐up parameters, derived from MODIS data, for multiple mixed oak and beech forest stands (Soudani et al. 2008). It was shown that the inflexion point of the piece‐wise logistic curve fitted to VI data (i.e., the point of maximum rate of change) was the best indicator of the onset of oak and beech bud‐burst in deciduous woodlands. This measure was also influenced less by understory phenology than the date of onset of VI increase (Soudani et al. 2008). In this study, we used GAMs to calculate a very similar metric, which were selected because we did not want to impose any prior assumption on the shape of the green‐up curve.

Interestingly, we found that the spatial patterns of pixel green‐up dates varied considerably from year to year (see Figs. 4 and S3). Even though the spring phenology of individual trees and shrubs is likely to be repeatable (Crawley and Akhteruzzaman 1988; Wesołowski and Rowiński 2006), the spring green‐up of different tree species is influenced by different environmental factors (e.g., winter chilling, photoperiod), and therefore, species will not always green‐up in the same order relative to each other (Roberts et al. 2015). In mixed species woodlands, this will likely lead to complex spatial patterns of green‐up that vary across years. Differences between years may also arise due to variation in spring cloudiness, which could conceivably influence the reliability of estimates of green‐up (see Discussion below).

### The problem of scale mismatch

The question of how variation in life‐history traits and ecological interactions is dependent on the scale at which it is studied is a key issue in ecology (Levin 1992). A recent study in topi antelopes (*Damaliscus korrigum*) found that the correlation between animal density and satellite‐derived primary production was dependent on the spatial resolution of the vegetation data (Bro‐Jørgensen et al. 2008). To date most studies that explore the relationship between bird, oak and caterpillar phenology have performed so at the population scale, where the environment is treated as homogenous across the population (e.g., Perrins 1965; van Noordwijk et al. 1995; Charmantier et al. 2008). Taking an approach that considers the scale at which individuals experience their environment is likely to be key if we are to understand the ecological and evolutionary processes acting on reproductive phenology within populations. Only through gaining this knowledge will we understand the scope for populations to cope with rapidly changing environments (Chevin et al. 2010). Recent work, that takes this individual‐based approach, has demonstrated that marked spatial variation in oak and caterpillar phenology exists at a scale relevant to individual birds and that great tits are able to fine tune their reproductive phenology to their immediate environment, by responding to environmental cues experienced over a relatively smaller spatial scale (i.e., within tens of meters of the nest) (Hinks et al. 2015). This demonstrates the power of measuring environmental variation at an appropriate scale to reveal processes masked by population‐level studies. Here, we found similar fine‐scale within‐population spatial variation in timing of breeding, where nestboxes within a few hundred meters of one another were more phenologically synchronized than those further apart. Furthermore, the scale of spatial autocorrelation appears to differ across the population, with timing being relatively homogeneous in some areas of the wood and spatially heterogeneous in others (see Fig. 3). These complex patterns of variation are likely to reflect similar spatial autocorrelation in the phenology of tits' food source (Hinks et al. 2015) and resource availability (Wilkin et al. 2007).

In this study, we use vegetation green‐up date with a spatial resolution of 240 m. (*c*. 5.8 ha). While this is still significantly larger than the average territory of a breeding great tit or blue tit (*c*. 1–3 ha, Stauss et al. 2005), meaning that there is still a degree of scale mismatch, it is likely to be a more ecologically relevant than environmental measurements estimated at the population scale (385 ha). By analyzing variation at this spatial scale, we found that the predictive power of satellite‐derived green‐up data to explain variation in timing of breeding varied across the woodland. In both tit species, neighboring pixels were significantly more similar to one another in terms of strength of phenological synchrony than those further apart. In blue tits, this spatial structure was more complex, with positive autocorrelation occurring at multiple spatial scales. At the pixel scale, blue tits were significantly more synchronized with vegetation green‐up than great tits. This may suggest that there are differences between the two species in the cues used to time laying, or in the scale that these cues are experienced.

We found that the extent to which great tits and blue tits matched the phenology of the local vegetation was related to habitat type. Pixels where birds were well synchronized with the vegetation green‐up were also those with high oak tree density. In contrast, in very oak‐poor areas of the woodland birds showed little or no synchrony with this measure of vegetation phenology. In Wytham, oak trees support the great majority of caterpillar biomass (Feeny 1970; Wint 1983) and great tit and blue tits are rarely seen foraging on beech, ash, or sycamore (Gibb 1954). It is therefore not surprising that birds nesting in oak‐poor areas are not synchronizing their breeding with the vegetation phenology measures we detect via remote sensing (which will be largely driven by nonoak species). Instead, we might expect that their laying date may covary with the phenology of the few oak trees in the vicinity of their nest, which account for too small a percentage of overall canopy to be contribute significantly to the EVI2 signal. Alternatively, their timing may be synchronized with oak‐rich areas further from the nest (i.e., outside the pixel containing their nestbox). Oaks are a key component of great tit habitat; tits nesting on oak‐rich habitat tend to breed earlier, feed their young on a higher proportion of caterpillars and have higher reproductive success (Wilkin and Sheldon 2009). It is therefore possible that the relationship we find between phenological synchrony of oak density is to some extent driven by good quality birds, which may be better at timing their breeding, settling in oak‐rich areas.

The relationship between phenological synchrony and oak prevalence was present in both tit species. Hazel was also a strong predictor of matching in great tits, but a much weaker predictor in blue tits. In early spring, great tits and blue tits vary in their foraging behavior; great tits are mainly observed feeding on and around hazel trees and then move to oak later in the spring, whereas blue tits favor oak throughout the spring and are considered the more specialized of the two species (Gibb 1954; Stauss et al. 2005). Our results are in agreement with these observations as the amount of hazel in a pixel covaried with phenological matching in great tits (for whom hazel is a critical component of foraging habitat) but less so in blue tits (for whom hazel is far less important). Taken together, these results suggest that using satellite data with a 240‐m resolution may only be useful where the satellite‐derived green‐up signal is driven predominantly by the type of vegetation used by foraging tits (e.g., homogenous oak stands), rather than oak‐ and hazel‐poor woodlands.

Intriguingly, green‐up dates for many of the MODIS pixels intersecting Wytham were in late March to mid‐April, before canopy species, such as oak, come into full leaf (see Figure S3). Similarly, a trend for earlier blue tit egg laying dates to be associated with pixels with higher NDVI values was noted at dates when oaks were still dormant in Southern France (Szulkin et al. 2015). This suggests that a significant amount of the green‐up signal may be caused by understory or ground‐level vegetation. However, the fact that satellite‐derived green‐up date can be used to predict timing of breeding tits, particularly in oak and hazel‐rich habitats, suggests that these metrics are providing biologically meaningful information about vegetation phenology. Further work is clearly needed to understand precisely which factors contribute to the remotely sensed phenological signal detected in different habitat types. Simply ground‐truthing satellite data using phenological data for individual trees and plants are likely to be logistically very difficult. At the level of individual trees, there is limited spatial autocorrelation in green‐up (E. F. Cole & B. C. Sheldon, unpubl. data), meaning that ground truthing may require sampling hundreds or thousands of trees and shrubs. One potential approach could be to use a combination of detailed habitat data and high‐resolution green‐up data collected by unmanned aerial vehicles (UAVs) to better determine species‐specific green‐up signatures in mixed woodlands.

In this study, we used satellite data with the highest spatial resolution available (given the additional requirement for high temporal resolution) for quantifying vegetation phenology in Wytham. The relatively coarse‐grained nature of these data limits the ability to analyze phenological synchrony at the finest scale. While satellite data with high spatial resolution do exist (e.g., Landsat7 up to 15 m resolution), the temporal resolution is too low to retrospectively quantify spring green‐up. It may be possible to increase the spatial resolution of MODIS data using a swath cumulation technique, which uses the finer scale geo‐referencing of the ungridded data from the MODIS sensors (Packalén and Maltamo 2007; Scambos et al. 2007), and by combining satellite data from multiple sources. Another potential method of obtaining high‐resolution remotely sensed vegetation phenology data is via frequent surveys using UAVs. These devices are becoming increasingly affordable and have an extremely high spatial resolution (e.g., subdecimeter) (Chabot and Bird 2013). As technology improves, these types of devices are likely to play an important role in the study of spatial ecology (Anderson and Gaston 2013).

### Sources of error in satellite data

Satellite data can suffer from multiple sources of error caused by atmospheric and ground conditions and sensor resolution and calibration (Pettorelli et al. 2005). MODIS EVI2 datasets, in addition to many similar products, have been quality‐controlled and preprocessed to minimize these types of error. However, one factor that limits the reliability of all satellite‐derived multispectral data is the amount of cloud cover. Cloudiness varies considerably across both time and space, with the UK experiencing higher than average cloudiness relative to continental Europe. We used MODIS cloud fraction data to show that early spring cloudiness, which differs between years (see Figure S4), relates to our ability to estimate vegetation green‐up dates from MODIS data. In years with high levels of cloudiness during February and March, we found little or no correlation between individual laying date and nestbox‐specific green‐up, in both great tits and blue tits. However, when early spring cloudiness was low, a much stronger relationship between vegetation and tit phenology was detected. This is likely to reflect the fact that in cloudy years, our estimates of green‐up are less reliable because green‐up trajectories will be based on a smaller number of clear images. Cloud cover is therefore a significant limitation to using satellite data to study animal phenology, and results based on these data should always be interpreted with care.

It is encouraging that, despite a mean cloudiness of 79% in January–March at the study site (see Figs. 6, S4 and S5), the remotely sensed signal of vegetation green‐up was sufficiently strong to reveal significant relationships with animal phenological traits. The pixel‐wise approach taken in this study, where green‐up measures for each 8‐day period are calculated by averaging multiple observations, means that despite high average cloud cover, it is still possible to obtain enough clear looks to estimate a time series of EVI2 for each pixel. Clearly the more cloud‐free observations, the more accurate the estimate of green‐up date will be. Given that Wytham has higher than average cloudiness with respect to the rest of Europe, this study suggests that the potential for using satellite data in predicting small‐scale phenological variation in other long‐term studies in temperate regions may be even greater.

## Conclusion

The aim of this study was to explore the utility of freely available remotely sensed satellite data for predicting small‐scale phenological variation in breeding behavior of birds. This investigation revealed that vegetation green‐up date related to timing of laying and hatching at the individual level, and the predictive power of this measure varied over space, depending on habitat type. Vegetation phenology varied considerably within the study site, and these spatial patterns varied between years. The existence of this small‐scale variation suggests that selection should favor individuals that respond to cues within their immediate environment in order to match their phenology to that of their food source. Knowledge of the environmental cues organisms use to time their breeding, and the scales at which these cues are experienced, is likely to be central to understanding how selection acts on these phenological traits.

To our knowledge, this is the first study to demonstrate spatial variability in synchrony within a single population. That this variability is related to habitat composition suggests that some individuals may be responding to cues from their environment, other than those closely linked to pixel‐level vegetation phenology (as detected by remote sensing). In fact, we find that tit and vegetation phenology are most synchronized in woodland areas dominated by the tree species in which great tits and blue tits predominantly forage. Not only does this add credence to the suggestion that the satellite‐derived phenology data is biologically meaningful at this scale, it also provides insight into small‐scale trophic interactions. Such marked within‐population variation in the extent to which phenology of different trophic levels match suggests that more attention should be given to studying environmental variation at a scale relevant to individual animals when exploring the causes and consequences of phenological synchrony.

To conclude, this promising approach to studying within‐population variation in phenology not only reduces the expense and manpower needed to quantify vegetation phenology, but also provides a standardized measure that could be used across studies. This approach will prove more informative for study sites where vegetation is well characterized. More work is clearly needed to understand which vegetation signals are driving remotely sensed green‐up in different habitats. These data do have great potential as they can be obtained in retrospect and therefore can be coupled with long‐term datasets to assess trophic interactions over time. As a result, this approach could greatly broaden the scale and scope of studies exploring phenological synchrony between trophic levels. The use of satellite‐derived data is now commonplace in conservation and population‐level ecological studies and has clear potential for exploring more small‐scale processes.

## Data accessibility

We provide the dataset via the Dryad repository (doi:10.5061/dryad.7v1qg).

## Conflict of Interest

None declared.

## Supporting information


**Figure S1.** Wytham habitat compartments (*n* = 121).
**Figure S2.** MODIS pixels (240 m × 240 m) that intersect with Wytham Woods (*n* = 117).
**Figure S3.** Spatial variation in spring vegetation green‐up date (2001–2013), measured as the first inflection point in Enhanced Vegetation Index 2 (EVI2), derived from a time series of MODIS satellite images for each pixel (*n* = 117).
**Figure S4.** Boxplot representing variation in the Wytham cloud fraction (in %, MODIS sensors) from January to March for years 2001–2013.
**Figure S5.** Boxplot representing variation in monthly cloud fraction in Wytham for years 2001–2013.
**Table S1.** Model outputs from Linear Mixed Models testing the relationship between individual laying date and hatch date and nestbox‐specific green‐up date for (a) great tits and (b) blue tits.
