# Supplementary material for: Predicting bird phenology from space: satellite‐derived vegetation green‐up signal uncovers spatial variation in phenological synchrony between birds and their environment
Source: Ecol Evol. 2015 Oct 19;5(21):5057–74. doi: 10.1002/ece3.1745 (PMC4662320; doi:10.1002/ece3.1745)
Supplement: Supplementary file 1 — Figure S1. Wytham habitat compartments (n = 121). Figure S2. MODIS pixels (240 m × 240 m) that intersect with Wytham Woods (n = 117). Figure S3. Spatial variation in spring vegetation green‐up date (2001–2013), measured as the first inflection point in Enhanced Vegetation Index 2 (EVI2), derived from a time series of MODIS satellite images for each pixel (n = 117). Figure S4. Boxplot representing variation in the Wytham cloud fraction (in %, MODIS sensors) from January to March for years 2001–2013. Figure S5. Boxplot representing variation in monthly cloud fraction in Wytham for years 2001–2013. Table S1. Model outputs from Linear Mixed Models testing the relationship between individual laying date and hatch date and nestbox‐specific green‐up date for (a) great tits and (b) blue tits. [file ECE3-5-5057-s001.docx]

**SUPPLEMENTARY MATERIAL**

**Supplementary Methodology**

**Processing of satellite data**

We used a time-series of level 3 gridded data, using products MOD09Q1 and MYD09Q1. These data are 8-day surface reflectance in red and near infrared, gridded at 7.5 arcsec resolution. All pixels from the h17v03 10-degree tile covering Wytham (n=117, see supplementary Fig. 2) were projected to 240m resolution on WGS 1984 UTM 30N coordinate system.  The Enhanced Vegetation Index 2 (EVI2) (Jiang et al. 2008) was then calculated for each pixel in each 8-day period. EVI2 was chosen because, like EVI, it performs better than NDVI in areas of dense vegetation, as it does not become saturated. EVI and EVI2 have been shown to be almost identical while EVI2 can be calculated without the use of a lower spatial resolution blue band. This allows greater independence in the EVI2 of adjacent pixels when EVI2 is calculated at 240m resolution.  A Generalised Additive Model (GAM; Hastie & Tibshirani 1990) was then fitted to the EVI2 data for each pixel individually (EVI2 vs. day of year). The day of vegetation green-up for each pixel was calculated as the day on which the derivative of this function was maximized (i.e. the day of the greatest rate of change in green-up, see Fig. 1). GAMs were fitted using the R package ‘mgcv’ version 1.8-4 and differentiated using the function predict.gam of mgcv (Wood 2011).

Nestbox-specific green-up dates were calculated using a weighting procedure to account for where in a pixel a nestbox was located as well as whether a pixel contained non-woodland. This weighting considered the four nearest pixels to each nestbox and weighted the estimated day of green-up based on the reciprocal of the distance between the nestbox and the centroid of each pixel.  If any of the four nearest pixels intersected the perimeter of the woodland, values were further weighted accounting for the proportion of each pixel that was comprised of woodland. These values were calculated using ArcGIS 10.2 software (see Supplementary Fig. 2).


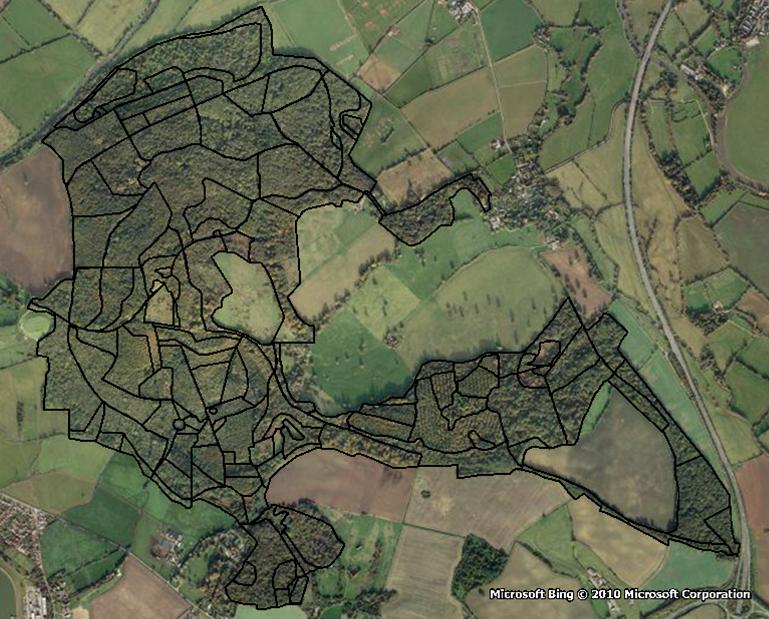


Supplementary Figure 1. Wytham habitat compartments (n=121).


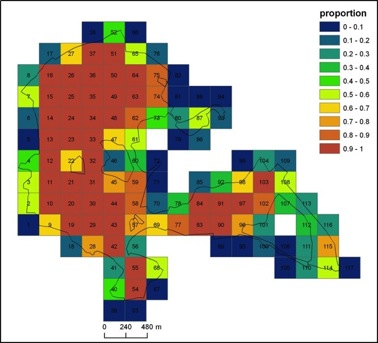


Supplementary Figure 2. MODIS pixels (240m × 240m) that intersect with Wytham Woods (n=117). Pixels are colour-coded based on the proportion of the pixel that falls inside the woodland. Number of pixels that contained >50% woodland = 67.

Supplementary Figure 3. Spatial variation in spring vegetation green-up date (2001 – 2013), measured as the first inflection point in Enhanced Vegetation Index 2 (EVI2), derived from a time series of MODIS satellite images for each pixel (n =117).

Supplementary Figure 4. Boxplot representing variation in the Wytham cloud fraction (in %, MODIS sensors) from January to March for years 2001-2013. Black vertical bars and red dots represent median and mean values, respectively. The red vertical line represents the average cloud fraction for the entire dataset.

Supplementary Figure 5. Boxplot representing variation in monthly cloud fraction in Wytham for years 2001-2013. Black vertical bars and red dots represent median and mean values, respectively.

Supplementary Table 1. Model outputs from Linear Mixed Models testing the relationship between individual laying date and hatch date and nestbox-specific green-up date for (a) great tits and (b) blue tits.

| **(a) GREAT TIT** | **LAYING DATE (3775)** | | | | **HATCH DATE (3656)** | | | |
| --- | --- | --- | --- | --- | --- | --- | --- | --- |
|  | coefficent | SE | t value | pMCMC | coefficent | SE | t value | pMCMC |
| itercept | -7.944 | 10.399 | -0.764 |  | 12.306 | 9.683 | 1.271 |  |
| green-up (within-year) | 0.016 | 0.007 | 2.278 | 0.020 | 0.019 | 0.007 | 2.919 | 0.002 |
| green-up (between year) | 0.325 | 0.124 | 2.616 | 0.028 | 0.337 | 0.116 | 2.914 | 0.006 |
| altitude | 0.025 | 0.004 | 6.929 | <0.001 | 0.026 | 0.003 | 7.379 | <0.001 |
| distance from woodland edge | 0.003 | 0.001 | 3.423 | <0.001 | 0.005 | 0.001 | 5.451 | <0.001 |
| local oak density | -0.038 | 0.006 | -5.936 | <0.001 | -0.031 | 0.006 | -4.962 | <0.001 |
|  |  |  |  |  |  |  |  |  |
| **(b) BLUE TIT** | **LAYING DATE (3744)** | | | | **HATCH DATE (3729)** | | | |
|  | coefficent | SE | t value | pMCMC | coefficent | SE | t value | pMCMC |
| intercept | -11.190 | 9.191 | -1.218 |  | 11.264 | 9.582 | 1.175 |  |
| green-up (within-year) | 0.013 | 0.008 | 1.664 | 0.076 | 0.011 | 0.007 | 1.671 | 0.088 |
| green-up (between year) | 0.344 | 0.110 | 3.128 | 0.014 | 0.345 | 0.115 | 3.009 | 0.012 |
| altitude | 0.038 | 0.004 | 10.144 | <0.001 | 0.039 | 0.003 | 11.984 | <0.001 |
| distance from woodland edge | 0.001 | 0.001 | 0.627 | 0.522 | 0.004 | 0.001 | 4.580 | <0.001 |
| local oak density | -0.065 | 0.006 | -11.433 | <0.001 | -0.044 | 0.005 | -8.896 | <0.001 |

Models control for the fixed effects: altitude, distance from the edge of the woodland and local oak density, and random effects: year and nestbox and female ID. NB: When nestbox-specific green-up date was tested alone it significantly related to both onset of laying and hatching date in both great tits (laying date: t=3.402, pMCMC=0.002, B ± SE=0·024 ± 0·007; hatching date: t=4.218, pMCMC<0.001, B ± SE=0·028 ± 0·007) and blue tits (laying date: t=2.929, pMCMC=0.008, B ± SE=0·023 ± 0·008; hatching date: t=3.374, pMCMC<0.001, B ± SE=0·023 ± 0·007).
